# The Care and Treatment of Exposed Pulps

**Published:** 1884-08

**Authors:** B. F. Luckey


### THE CARE AND TREATMENT OF EXPOSED PULPS.

BY B. F. LUCKEY, D. D. S.

Extract From a Paper Read Before the Central Dental Association of Northern New Jersey.

In attempting to preserve the pulp, the general health of the patient and the condition of the pulp must be taken into consideration. If the general systemic condition is good, or fairly good, and the exposure recent, the case may be treated and carried to completion at once; but if the patient is debilitated—anæmic—and the system run down, or if the pulp has been exposed for a long time, more care will be necessary, and more caution required in its treatment.

There have been many ways and methods of treatment pursued and advised for the salvation of the pulp. A great variety of materials have been recommended for cappings, prominent among which are sheet lead, gold plate, card-board, cork, cup-shaped platina caps, gutta-percha, oxy-chloride and oxy-phosphate of zinc, collodion, shellac, etc. Old Dr. Hullihen used a conical tube filled with morphine and creosote. A favorite method of treatment for a long time, and a very good one too, has been to cap the exposed portion with a thin mixture of oxide of zinc and creosote, and after absorbing the excess of creosote with a little spunk or bibulous paper, to partially fill the cavity with any of the oxy-phosphates or oxy-chlorides. It is the method I pursued for a number of years—but for some time past I have been treating exposed pulps in a way that has given me more complete satisfaction and a larger percentage of success than I have ever had by any other method.

In a case of a very recent or an accidental exposure, where there is little or no inflammation, after removing the decay from the cavity as thoroughly as the circumstances will admit, I wipe out the cavity gently with a pellet of bibulous paper saturated with a mixture of oil of cloves and creosote, which, coagulating the albumen of the organic tissue, forms at once a protecting coat for the exposed pulp, and obtunds the sensitiveness of the dentine ; I then place over the point of exposure a small quantity of iodoform paste, and over that I place a layer of asbestos felt, allowing it to lap well over on the solid dentine. I then cover that with oxy-phosphate of zinc mixed to the consistency of thick cream, commencing at the edges and working carefully toward the center, avoiding as much as possible all pressure over the exposed point; after the oxy-phosphate has hardened sufficiently, it may be trimmed enough to allow of permanent filling over it with gold or amalgam.

The formula for the iodoform paste is :

℞ Iodoform, ℥ss.

Balsam Peru., ʒi.

Tonka bean, (grated fine), q. s. M.

I find that the paste acts like a charm in reducing the tendency to inflammatory action to the minimum, and in soothing whatever irritation may be present ; the asbestos felt is much better for capping than the gutta-percha ordinarily used, in that it is, I believe, a more complete non-conductor of thermal changes ; it is more tractable under the instrument, easier of adaptation, and does not swell and thereby cause pressure on the pulp, as we know that gutta-percha sometimes does.

All this can be done and the operation completed in one sitting, with the assurance of success that I have never had with any other treatment. In cases of long exposure, with considerable inflammation, by a slight incision I first cause a free flow of blood to deplete the pulp and relieve the tension, and after the bleeding has ceased proceed as before, with this exception, that instead of oxy-phosphate I use a temporary stopping of gutta-percha and wax, so that in case of trouble it can be easily removed, and further treatment resumed as the case may indicate.

One of the severest tests I have ever given this treatment was in a superior left first molar, in the mouth of a rather delicate young woman. She had been suffering a great deal with the tooth, and on excavating I found both of the anterior horns of the pulp badly exposed, either one of the openings being large enough to admit the head of a large pin—in fact, I could see it pulsate. I treated it in the manner I have related, covering the asbestos with gutta-percha and wax. The pain ceased before she left the office, and after a trial of about two months, I removed the gutta-percha and substituted oxy-phosphate, finishing with amalgam, the tooth being alive and apparently doing well ; that was about four months ago. I saw her recently, and she declared that she had not had a twinge of pain since the first treatment of the tooth.

I have also succeeded in amputating a portion of the pulp ; sometimes as much as half of it, and with this same treatment saved the remainder; at least, I have good reason to presume they were saved, because they have given no further trouble.

In preparing cavities for filling, if there is much tenderness or a ready response to thermal changes, I always place in the bottom of the cavity a layer of asbestos felt as a precautionary measure to protect the pulp, and thereby save my patient much after pain and suffering, and myself much extra work.

In case a pulp becomes thoroughly inflamed I do not believe it is policy to attempt to save it. Death is almost inevitable, for the vessels become so engorged with blood, especially the afferent vessels, that strangulation at the apicial foramen is occasioned. When the symptoms indicate that the pulp is thoroughly inflamed, I believe the safest plan is to devitalize and remove it as soon as possible

